# Traditional and New Views on MSI-H/dMMR Endometrial Cancer

**DOI:** 10.3390/biom15101370

**Published:** 2025-09-26

**Authors:** Chuqi Liu, Huiyu Ping, Mengmeng Yao, Xinru Li, Qingxin Li, Ruotong Hu, Yawen Xu, Kaidi Meng, Fei Gao, Kai Meng

**Affiliations:** 1 Lin He’s Academician Workstation of New Medicine and Clinical Translation, Jining Medical University, Jining 272067, China; liuchuqi@stu.mail.jnmc.edu.cn (C.L.); pinghuiyu@stu.mail.jnmc.edu.cn (H.P.); yaomengmeng@stu.mail.jnmc.edu.cn (M.Y.); lixinru@stu.mail.jnmc.edu.cn (X.L.); liqingxin@stu.mail.jnmc.edu.cn (Q.L.); huruotong@stu.mail.jnmc.edu.cn (R.H.); xuyawen@stu.mail.jnmc.edu.cn (Y.X.); 2 College of Clinical Medicine, Jining Medical University, Jining 272067, China; 3 College of Medical Imaging and Laboratory Medicine, Jining Medical University, Jining 272067, China; 4 College of Medical Laboratory, Qilu Medical University, Zibo 255300, China; mengkaidi@qlmu.edu.cn; 5 Institute of Zoology, Chinese Academy of Sciences, Beijing 100101, China

**Keywords:** MSI-H/dMMR endometrial cancer, base mismatch repair pathway, next-generation sequencing

## Abstract

MSI-H/dMMR endometrial cancer (EC) is closely linked to the mismatch repair (MMR) pathway, and its pathogenesis is associated with microsatellite instability (MSI) caused by abnormalities in the core genes of the conventional MMR system. This cancer exhibits a distinct immune microenvironment, which makes it suitable for treatment with immune checkpoint inhibitors (ICIs). This cancer type demonstrates heterogeneity, encompassing Lynch syndrome (LS)-associated EC (characterized by germline mutations), sporadic EC (attributed to *MLH1* promoter hypermethylation), and Lynch-like EC (driven by somatic mutations). Research indicates that these three dMMR EC subtypes possess different immune microenvironments, which may influence the therapeutic efficacy of ICIs. However, the impact of somatic mutations in traditional MMR genes on EC has often been overlooked. Furthermore, over 50% of patients with MSI exhibit no response to ICIs, potentially due to abnormalities in nontraditional MMR genes. This review discusses the role of traditional and nontraditional MMR genes in dMMR EC and related treatment strategies, highlights key issues in the current diagnosis and treatment of dMMR EC, and aims to enhance understanding of its heterogeneity and advance precision diagnosis and treatment.

## 1. Introduction

Endometrial cancer (EC), also known as uterine body cancer, is the fourth most common gynecologic malignancy in developed nations [1]. According to 2024 cancer statistics, the incidence of EC is rising by approximately 2% annually, while its mortality rate is increasing more rapidly than that of breast, cervical, and ovarian cancers [1,2]. EC traditionally occurs in postmenopausal women, but recent clinical data indicate that it is increasingly diagnosed in younger women [3]. Pathologically, EC is classified as either endometrioid endometrial carcinoma (EEC) or non-endometrioid carcinoma. EEC is typically an early-stage, low-grade tumor that is primarily driven by elevated levels of endogenous or exogenous estrogen [4]. In contrast, non-endometrioid carcinoma encompasses highly invasive, high-grade tumors such as clear cell endometrial carcinoma (CCEC) and uterine serous carcinoma (USC), along with other rarer subtypes [5], and its pathogenesis is unrelated to estrogen exposure. The rising mortality rate of EC is associated with an increased incidence of advanced high-grade tumors [6]. Lynch syndrome (LS) is a common hereditary risk factor of hereditary EC. It is also known as hereditary non-polyposis colorectal cancer (HNPCC). It is an autosomal dominant genetic disease disorder caused by germline mutations in the mismatch repair (MMR) genes *MLH1*, *MSH2*, *MSH6*, and *PMS2* [7]. Individuals with LS have an elevated lifetime risk of developing cancer. Research indicates that approximately 40–60% of patients with LS develop their first malignant tumor as EC [8]. Currently, EC associated with LS is mainly classified as EEC [9]. Histological evaluation is crucial for prognostic stratification and treatment. However, reliance solely on histological evaluation presents challenges, including overlap between subtypes, low reproducibility of grading, and poor interobserver diagnostic consistency, which may lead to either insufficient or excessive treatment [10]. In 2013, The Cancer Genome Atlas (TCGA) reported that non-endometrioid carcinoma and 25% of high-grade EECs exhibit extensive somatic copy number alterations, frequent *TP53* mutations, and other molecular characteristics, whereas most EECs demonstrate microsatellite instability (MSI). Therefore, TCGA classifies EC into four molecular subtypes: *POLE* hypermutated, microsatellite instability-high (MSI-H), high copy number/abnormal p53, and low copy number/wild-type p53. This classification will facilitate the development of precision therapies for EC based on molecular profiling [11].

Normal cells rely on the MMR system to maintain genomic stability. When MMR function is deficient (dMMR), MSI can occur [12]. In EC, the detection of MSI-H and dMMR yields highly consistent results [13,14]. Therefore, in most literature, the terms ‘dMMR’, ‘MSI-H’, and ‘MSI-H/dMMR’ are used interchangeably to denote the same molecular subtype characterized by MMR defects. MSI typically manifests as the accumulation of insertion and deletion errors in microsatellite sequences during DNA replication, which significantly increases the frequency of frameshift mutations in the coding region [15]. This accumulation of mutations can increase the tumor mutation burden (TMB), promote the production of numerous novel antigens, and enhance tumor-infiltrating lymphocytes (TILs) [16,17]. These novel antigens serve as key targets for immune recognition of tumors and initiation of T cell-mediated antitumor immune responses [18]. Therefore, patients with MSI-H/dMMR ECs benefit from immune checkpoint inhibitor (ICI)-based immunotherapy. It is important to highlight that MSI-H/dMMR ECs account for approximately 25% to 30% of all EC cases [19]. This category of EC arises from three distinct pathways: LS-related EC, which develops from the LS; sporadic EC, which results from hypermethylation of the *MLH1* promoter; and Lynch-like EC, which arises from somatic mutations in *MLH1*, *MSH2*, *MSH6*, and *PMS2*. Evidence from limited studies indicates variations in the immune microenvironment across these three subtypes of dMMR tumors, as well as differences in their response to identical immunosuppressive agents. These findings underscore the importance of accurately distinguishing the three subtypes to guide treatment strategies effectively [20]. Nevertheless, Lynch-like EC is frequently overlooked in the existing literature and in routine clinical screening practices. Most research and clinical focus is directed towards hereditary and sporadic EC, which account for approximately 5% and 70% of cases, respectively [21,22]. Although current guidelines from the National Comprehensive Cancer Network (NCCN) recommend immunohistochemistry (IHC) of four key proteins, followed by germline testing to identify LS, and methylation analysis to confirm *MLH1* methylation, ICIs are recommended based on pooled populations with dMMR. Furthermore, studies indicate that more than 50% of patients with EC and MSI-H do not respond to ICIs [23]. Considering that studies have identified abnormalities in other MMR genes, such as *MLH3*, in patients [24,25,26], we speculate that these abnormalities may involve additional genes associated with MSI within the MMR pathway.

Indeed, the intrinsic high-frequency and cyclical proliferation of endometrial cells highlights the critical role of the MMR pathway in preserving genomic stability within endometrial tissue throughout its normal cyclic transformations [27]. As a result, dysfunction in the MMR pathway hinders the correction of base–base mismatches occurring during DNA replication. This impairment leads to MSI and elevates the error rate in genes associated with cancer, thereby facilitating cellular carcinogenesis [28,29]. This process constitutes a fundamental pathogenic pathway in uterine carcinogenesis and may also be instrumental in the development of other gynecological malignancies exhibiting endometrioid characteristics [30,31]. For example, an increasing body of evidence indicates that the molecular mechanisms characterized by dMMR and MSI are linked to the development of various extrauterine tumors associated with endometriosis. While endometriosis is typically regarded as a benign condition, it retains a potential for malignant transformation, with an incidence rate estimated to be between 0.7% and 1.6% [32,33]. Santoro et al. documented an uncommon case involving a 62-year-old female patient who was simultaneously diagnosed with clear cell ovarian carcinoma and low-grade endometrial stromal sarcoma, both neoplasms arising from endometriotic lesions. This case further substantiates the hypothesis that endometriosis can act as a potential precursor for various types of gynecologic malignancies [33]. In addition, Yamaguchi et al. reported two cases of Lynch syndrome in which immunohistochemical analysis demonstrated a loss of MSH2 and MSH6 protein expression in endometrial cancer, ovarian cancer, and adjacent endometriotic tissues. These findings suggest that a deficiency in MMR proteins may facilitate the progression of ovarian endometriosis to ovarian cancer [34]. In a patient with a documented history of atypical endometrial hyperplasia and ovarian endometriosis, clear cell carcinoma of the diaphragm was diagnosed three years following the completion of total hysterectomy and bilateral salpingo-oophorectomy. Genetic testing revealed pathogenic mutations in the germline of the *MSH2* gene. The tumor tissue demonstrated MSI-H, and immunohistochemical analysis indicated a loss of MSH2 and MSH6 protein expression [32]. This suggests that dMMR/MSI may act as a cross-anatomical driver with broad pathogenic relevance in tumors of endometrioid phenotype.

This review aims to systematically explore more comprehensive research progress and related treatment strategies concerning the traditional core genes (*MLH1*, *MSH2*, *MSH6*, *PMS2*) and auxiliary genes (*MLH3*, *MSH3*, *EXO1*) of the MMR pathway in EC, hoping to provide new and comprehensive insights for the future diagnosis and treatment of EC, thereby contributing to the improvement of survival rates and quality of life for EC patients. However, readers should be aware of the broader systemic implications of this mechanism in gynecologic oncology.

## 2. The Mechanism of MMR Pathway and MSI-H/dMMR EC

**Figure 1:**
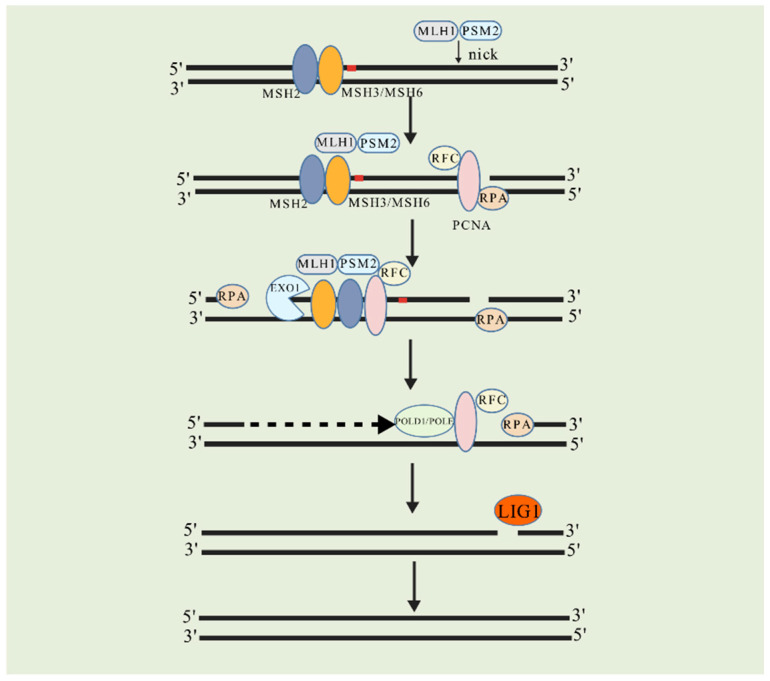
Human MMR. MSH2-MSH6 and MSH2-MSH3 heterodimeric complexes recognize mismatched bases, thereby initiating the MMR process. Following this recognition, the MLH1-PMS2 complex is recruited, leading to an incision downstream of the site of the mismatch site. Subsequently, RFC facilitates the loading of PCNA at the incision site. EXO1 excises the erroneous DNA fragment, while RPA binds to and stabilizes the exposed ssDNA. The resultant gap is synthesized by POLD1 or POLE. Finally, LIG1 seals the remaining nicks, completing the repair process. Created with BioGDP.com [43].

In eukaryotes, MMR maintains genomic stability by correcting base-base and small insertion–deletion mismatches generated during DNA replication through the coordinated action of multi-protein complexes (Figure 1). The MutS complex facilitated the initiation of MMR. Specifically, the MSH2–MSH6 heterodimer (MutSα) recognizes single-base mismatches, such as thymine-guanine (T-G) repairs, as well as one- to two-nucleotide insertions or deletions. In contrast, the MSH2–MSH3 heterodimer (MutSβ) specializes in recognizing larger mismatched fragments ranging from 2 to 16 nucleotides [35]. Subsequently, MutL complexes, such as MLH1–PMS2 (MutLα), are recruited and introduce incisions downstream of the mismatched site through their endonuclease activity, providing a directional signal for subsequent resection [36]. The proliferating cell nuclear antigen (PCNA) functions as a “sliding clamp” during DNA replication. It enhances mismatch-recognition efficiency by binding to MutSα/β [37]. Additionally, PCNA collaborates with replication factor C (RFC) to load onto the DNA incision site, thereby activating the proofreading function of exonuclease EXO1 or DNA polymerase δ/ε [38]. EXO1 remove erroneous nucleotide sequences through synergistic interaction with PCNA and MutSα/β, resulting in the formation of a single-stranded DNA (ssDNA) gap. Concurrently, replication protein A (RPA) binds to and stabilizes the exposed ssDNA, protecting it from degradation by nonspecific nucleases [36]. Notably, the activity of EXO1 is modulated by MutSα, which not only activates the 5′-3′ exonuclease activity of EXO1 in a mismatch-dependent manner but also limits excessive cleavage by inducing conformational changes in the ATPase domain of MLH1, thereby preventing unnecessary genomic damage [39]. The gap created by this process is subsequently filled by DNA polymerases, specifically DNA polymerase δ (POLD1 subunit) and DNA polymerase ε (POLE subunit), which utilize the undamaged DNA strand as a template. Starting from the 3′ end of the incision, these polymerases synthesize new DNA fragments in accordance with the principle of complementary base pairing to fill the gaps left by the excision of mismatched nucleotides [40,41]. Finally, DNA ligase I (LIG1) seals the incision, restoring the continuity of the DNA double helix [42].

**Figure 2:**
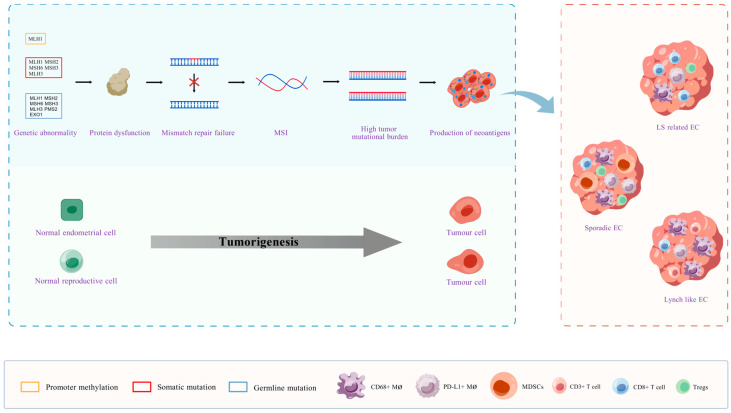
The pathogenic mechanism and immune microenvironment characteristics of MSI/dMMR EC. DNA MMR gene mutations or MLH1 promoter methylation can cause deletion or dysfunction of MMR proteins. This MMR system defect will hinder the effective correction of replication errors, leading to MSI, which in turn causes abnormal cell proliferation and carcinogenesis. MSI can further increase the tumor mutation burden (TMB) and promote the production of numerous novel antigens, thereby affecting the anti-tumor immune response. Ls-related EC, sporadic EC and Lynch-like EC exhibit distinct tumor microenvironments. In LS-related EC, the number of CD8+ T cells in the stroma is high, while CD68+ and PD-L1+ macrophages in the stroma and tumor compartments are few, resulting in strong immunogenicity. In sporadic EC, the number of CD8+ T cells in the matrix is relatively small, while the number of CD68+ macrophages and PD-L1+ macrophages in the matrix and tumor compartments is relatively large, and the infiltration of CD3+ T cells is relatively low. The CD8+ T cells of Lynch-like EC are relatively few, the infiltration of CD3+ T cells shows an increasing trend, and the infiltration of CD68+ macrophages is significantly higher. Created with BioGDP.com [43].

DNA microsatellites, which consist of tandem repeats of 1–6 nucleotides, are distributed throughout both the coding and non-coding regions of the genome and are characterized by high mutation rates. MMR plays a key role in regulating microsatellite length, and its defects can cause uncorrected duplication errors, resulting in abnormal repetitions and, ultimately, the development of MSI [44]. Traditionally, germline mutations in key MMR genes, including *MLH1*, *MSH2*, *MSH6*, and *PMS2*, as well as hypermethylation of the *MLH1* promoter, have been thought to contribute to dMMR, thereby inducing MSI and subsequent carcinogenesis [45]. However, emerging evidence suggests that beyond these classical mechanisms, somatic mutations in the aforementioned genes, as well as germline or somatic alterations in non-canonical MMR genes such as *MSH3*, *MLH3*, and *EXO1*, may similarly induce dMMR status and contribute to the development of MSI-H/dMMR endometrial carcinoma. These abnormalities in non-canonical MMR genes can compromise mismatch repair function through multiple mechanisms—such as impairing the nuclease activity of the genes themselves, disrupting their interaction with core MMR proteins like MSH2 or MLH1, or affecting the stability of the repair complex—ultimately leading to the accumulation of replication errors and MSI, thereby promoting endometrial carcinogenesis in either concerted or independent manners (Figure 2).

## 3. MMR Genes and MSI-H/dMMR EC

### 3.1. MSH2

*MSH2* is located on human chromosome 2p21-p16.3, and its encoded protein is a core protein in the MMR pathway. As previously noted, germline mutations in this gene cause dMMR, which in turn leads to MSI-type EC. Patients with *MSH2* germline mutations account for approximately 32% of all LS patients [46]. These mutations significantly increase the risk of EC in patients with LS. According to the study by Møller et al., the cumulative incidence of EC in LS patients carrying *MSH2* germline mutations was as high as 51% at the age of 70, ranking first among the four MMR genes of *MLH1*, *MSH2*, *MSH6*, and *PMS2*, and significantly higher than *MLH1* (34%) and *PMS2* (24%) [46]. Furthermore, previous studies have reported that the cumulative risk of EC in carriers of *MLH1* or *MSH2* germline mutations ranges from 14% to 54% [47]. Broaddus et al. examined 50 patients with LS-associated EC and identified seven cases of CCEC, uterine serous carcinoma (USC), and uterine carcinosarcoma attributed to *MSH2* germline mutations [48]. These tumors are primarily attributed to MSI and the resulting genomic instability induced by *MSH2* mutations. *MSH2* somatic mutations are also associated with Lynch-like EC. Chapel et al. employed next-generation sequencing (NGS) technology to identify somatic mutations in *MSH2* in a pair of atypical hyperplasia/endometrial intraepithelial neoplasia and EEC cases [49]. Riedinger et al. identified somatic mutations c.1165C > T (p.Arg389Ter), c.2246A > C (p.Glu749Ala), c.2168C > T (p.Ser723Phe), and c.1007del (p.Pro336fs) of *MSH2* in two cases of EEC and identified the somatic mutation c.2011A > G (p.Asn671Asp) of *MSH2* in one case of USC [50]. Mensenkamp et al. analyzed 25 MSI-positive tumors unexplained by germline mutations or promoter hypermethylation using ion semiconductor sequencing, including one case of EC caused by *MSH2* somatic mutations (c.562G > T and c.1600 del), without specification of pathological subtypes. As described above, *MSH2* somatic mutations can also cause EC by causing MSI [51]. In summary, both *MSH2* germline and somatic mutations causes loss of protein function, leading to dMMR, subsequent MSI, and genomic instability, ultimately driving tumorigenesis. Both germline and somatic mutations are associated with highly invasive histological subtype of EC.

### 3.2. MSH6

*MSH6* is located on human chromosome 2p15-16, and its encoded protein is one of the three most important MMR proteins in the MutS family [52], which mainly plays a role in the early stages of MMR. Germline mutations in *MSH6* are associated with MSI and dMMR EC. A prospective observational study reported that approximately 19.4% of all patients with LS carry a pathogenic *MSH6* mutation, with the cumulative prevalence of EC in 65-year-old carriers reaching 32.1% [53]. Furthermore, studies [54,55,56] have demonstrated that *MSH6* mutations account for 10–15% of all MMR gene mutation in LS-associated EC, and patients with *MSH6* mutations exhibit a higher risk of developing EC than colorectal cancer (CRC) [56]. Recently, Danley et al. identified a germline *MSH6* mutation, c.3848_3851del (p.I1283fs), in a case of USC [57]. Somatic mutations in *MSH6* are also prevalent in EC. Goodfellow et al. evaluated *MSH6* deficiency in a cohort of 100 patients with EC without distinguishing pathological subtypes [58]. They found somatic *MSH6* mutations in 17 patients, all of whom also exhibited MSI. In a separate study, Billingsley et al. analyzed 535 EECs using Sanger sequencing and identified somatic mutations in *MSH6* (c.1231G > C and c.890C > T) in two patients [59]. Walker et al. also identified an *MSH6* somatic mutation c.1135_1139del (p.Arg379*), in a well-differentiated FIGO stage 1 EEC using multi-gene panel sequencing [60]. A research group led by Dámaso E also identified an EC patient who was MSI-positive but retained MMR protein expression. Further genetic testing revealed a truncated *MSH6* mutation (c.2219T > A, p.Leu740*) which was confirmed to be pathogenic [61]. These suggests that *MSH6* somatic mutations may contribute to the development of Lynch-like EC by inducing MSI. Recent findings by Berg et al. demonstrated that beyond assessing MMR status, MSH6 protein expression levels can enhance the prognostic evaluation in EC. They reported that elevated *MSH6* and *MSH2* expression was significantly associated with invasive tumor characteristics, including high-grade EEC, non-endometrioid histological types, and advanced FIGO stages [62]. In conclusion, both germline and somatic *MSH6* mutations promote EC development by impairing MMR function, leading to MSI and genomic instability. Among these, somatic mutations are most strongly associated with Lynch-like low-grade EC. Furthermore, *MSH6* expression levels may serve as a prognostic marker for clinical stratification.

### 3.3. MSH3

*MSH3* is located on human chromosome 5q14.1 and encodes a protein that binds with MSH2 to form MutSβ [63], and shares overlapping functions with MSH6 in the MMR pathway [64]. The cooperative interaction between MSH3 and MSH6 may enhance the overall efficiency of MMR [65]. Although *MSH3* has not traditionally been a research focus, existing data suggest that *MSH3* abnormalities may impair MMR function, potentially causing MSI and subsequently contributing to EC development. Singh et al. identified five germline *MSH3* mutations in 199 EC cases (without distinguishing pathological types) by targeted sequencing, including c.1394A > G p.(Tyr465Cys), c.845C > T p.(Thr282Ile), c.1232G > A p.(Arg411His), c.1396A > G p.(Ser466Gly) and c.2041C > T p.(Pro681Ser). However, MSI was not further assessed in this study [66]. Duraturo et al. identified 13 germline *MSH3* mutations in 79 patients with LS. Notably, the c. 2732 T > G and c. 693 G > A mutations were detected in the proband of a patient who developed EC at the age of 63 years and was subsequently diagnosed with HNPCC. The patient also exhibited high MSI [65]. These findings suggest that germline *MSH3* mutations may contribute to the development of LS-related EC. In addition, *MSH3* mutations may also be associated with Lynch-like EC. Wang et al. suggested that MSH3 mutations are common in EC. They identified 50 cases of *MSH3* mutations in 562 patients with EC, including 35 cases of grade 3 EEC and 6 cases of USC [25]. However, this study did not distinguish between germline and somatic mutations. A family member of a patient with LS and endometrial hyperplasia was found to have a somatic frameshift mutation in *MSH3* [67]. In addition, in an earlier study, Risinger et al. found a somatic frameshift mutation (1148delA) of MSH3 in an MSI-positive primary EC (without distinguishing pathological types) and an MSI-positive cell line, HHUA, using a protein truncation test. They subsequently introduced chromosome 5, containing wild-type *MSH3* into the mutant HHUA cell line and found that MSI was partially restored, indicating that *MSH3* is involved in regulating MSI in human cells. The inactivation of *MSH3* may lead to genomic instability [68]. In yeast cells, *MSH2*-*MSH3* co-overexpression impairs MMR, leading to enhanced ATPase activity, MLH complex-dependent post-translational modification of PCNA, S-phase accumulation, and inhibition of DNA polymerase δ synthesis. These changes disrupt DNA metabolic pathways and cell-cycle progression, ultimately predisposing cells to genomic instability [69]. In summary, both germline and somatic *MSH3* mutations may cause MSI by disrupting MutSβ complex function, thereby leading to genomic instability. Germline mutations are linked to LS-associated EC, whereas somatic mutations are associated with highly aggressive EC. Moreover, *MSH3* overexpression can promote genomic instability through multiple mechanism and may represent a complementary pathway in the pathogenesis of EC.

### 3.4. MLH1

*MLH1* is located on the 3p22.2 region of the human chromosome, and its encoded protein interacts with PMS2 and MLH3 to form the MutLα and MutLγ complexes, respectively [70], which play an important role in MMR. Methylation of the *MLH1* promoter leads to loss of its encoded protein expression, and the resulting EC accounts for approximately 70% of dMMR EC [71,72,73], representing a confirmed epigenetic mechanism of sporadic EC [74]. An investigation of the relationship between *MLH1* promoter methylation status and MSI phenotype in 29 cases of sporadic EC revealed a significant association, suggesting that *MLH1* promoter methylation may be a key molecular mechanism driving the MSI phenotype in sporadic EC [75]. Manning-Geist et al. screened 184 patients with MMR-type EC among more than 1100 patients with EC and found that patients with sporadic EC were older, had higher body mass index, presented with later disease detection, and lower TIL scores than patients with EC harboring a germline or somatic MMR gene mutation, and that these differences may affect treatment and prognosis [76]. Furthermore, *MLH1* is the most prevalent MMR gene associated with LS, with germline mutations accounting for approximately 42% of LS cases [77]. Studies have shown that *MLH1* germline mutations can disrupt the MMR system, resulting in continuous accumulation of DNA replication errors in the microsatellite region and eventually causing MSI [78]. Patients with LS who carry *MLH1* germline mutations face a cumulative risk of developing EC by age 70 of approximately 54%, which is higher than the risk for other LS-associated cancers, such as CRC (41%) and ovarian cancer (20%) [79]. In contrast to sporadic EC, EEC is the predominantly pathological type associated with LS [48]. Momma et al. identified germline *MLH1* mutations in two patients with EC in a study of LS family members who were followed up for more than 45 years and exhibited a high frequency of MSI [80].

It is also noteworthy that pathogenic germline mutations in *MLH1* can coexist with *MLH1* promoter methylation, indicating that *MLH1* promoter methylation does not preclude a diagnosis of LS [81,82]. Somatic mutations in *MLH1* are a prevalent cause of dMMR in Lynch-like EC. Mensenkamp et al. detected 25 MSI-positive tumors using Sanger and ion semiconductor sequencing and identified the somatic *MLH1* mutation c.1252_1253del (p.Asp418fs) in EC [51]. Similarly, Mills et al. examined 210 patients with EC and found that EC was the initial malignancy in 87.5% of patients with Lynch-like syndrome (LLS), with high MSI levels observed in 71.4% of these patients [83]. Overall, abnormalities in *MLH1*, including methylation, germline mutations, and somatic mutations, lead to MSI by impairing MMR function, thereby contributing to the development of EC. Specifically, methylation predominantly occurs in sporadic EC and is linked to poor prognosis. Germline mutations are more frequently observed in EEC, and somatic mutations are the primary cause of Lynch-like EC.

### 3.5. MLH3

*MLH3* is located on the 14q24.3 region of the human chromosome and is broadly expressed in both embryonic and adult tissues. Early studies have indicated that the protein encoded by this gene associates with MLH1 to form the MutLγ complex, which primarily functions in meiosis [84,85]. Additional studies have demonstrated that the protein also performs a complementary role with PMS2 in the MMR pathway; however, its efficiency in repairing mismatches is notably lower than that of MutLα [86]. *MLH3* inactivation is associated with increased MSI in mammals [86,87]. Using NGS and Sanger sequencing in 274 patients with LLS, Xavier et al. identified *MLH3* germline mutations (c.885del, p.His296Thrfs*12), with this variant detected in one case of EC [88]. Korhonen et al. conducted a pioneering evaluation of the pathogenicity of seven *MLH3* germline mutations—including c.1939 C > T, c.2449 A > G, c.4351 G > A, and c.3826 T > C—identified in patients with CRC or EC, without differentiating between pathological types. Functional experiments demonstrated that while *MLH3* mutations alone do not impair MMR, they may elevate cancer risk through synergistic interactions with other MMR genes, such as *MSH2* mutations [89]. These findings suggest that *MLH3* is a promising candidate gene for LS, with germline mutations potentially increasing the risk of LS-associated EC through interactions with other genes. Furthermore, previous research indicated that 25% of MSI-H tumor samples exhibited *MLH3* somatic mutations in 36 cases of CRC [90], suggesting that such mutations may also contribute to the development of MSI-H tumors. In a study by Taylor et al. involving 57 patients with EC, 82.5% had EEC, 8.8% had mixed cell types, and 5.3% had CCEC. Nine germline mutations and three somatic mutations were identified. Notably, this study was the first to report that *MLH3* mutations may contribute to the development of EC. The authors suggested that this might be associated with genomic instability or disruptions in the apoptotic pathway caused by MSI due to *MLH3* mutations [24]. Wang et al. identified 30 cases of *MLH3* mutations (without distinguishing between germline and somatic mutations) in a cohort of 562 patients with EC. These cases included nine cases with only *MLH3* mutations and 21 in which *MLH3* mutations coexisted with other gene mutations. The tissue types affected by these mutations included EEC (grades G1–G3) and USC, with the highest rate of observed *MLH3* mutations in EEC G3 (14 cases) [25]. Additionally, Chang et al. analyzed WES-derived somatic mutation data from 531 EC samples in the TCGA pan-cancer map and found that 47 of 192 high dMMR-related mutation profiles harbored presumed somatic driver mutations in at least one MMR gene, including *MLH3*, *MSH2*, *MSH3*, and *MSH6* [91]. In a recent study, Saber et al. identified a missense *MLH3* mutation (g.75513463 A > G) in EEC at FIGO stage 1b using NGS [26]. These results indicate that somatic mutations in *MLH3* contribute to the development of Lynch-like EC by inducing MSI. Overall, both germline and somatic *MLH3* mutations contribute to the onset of EC. Germline mutations in *MLH3* may increase cancer susceptibility through synergistic interactions with other MMR genes, whereas somatic mutations in *MLH3* may trigger MSI independently, thereby facilitating the initiation and progression of Lynch-like EC.

### 3.6. PMS2

*PMS2* is located on the 7p22.1 region of the human chromosome. The protein encoded by PMS2 interacts with MLH1 to form a complex with endonuclease activity, which plays a pivotal role in the MMR pathway [36]. Earlier studies have suggested that patients with LS with *PMS2* germline mutations were relatively rare compared with mutations in other core genes, accounting for approximately 6% of all LS cases [92]. However, subsequent research demonstrated that the population frequency of *PMS2* germline mutation carriers is the highest among the four MMR genes, with reported frequencies of *MLH1* = 1 in 1946, *MSH2* = 1 in 2841, *MSH6* = 1 in 758, and *PMS2* = 1 in 714 [93]. Studies have indicated that individuals with *PMS2* germline mutations have a significantly higher risk of LS-related EC than CRC, and *PMS2* deficiency-related EC typically exhibits MSI-H characteristics [94]. A prospective study examining the expression of PMS2 protein in 3213 cases of CRC and 215 cases of EC (primarily EEC) revealed that 7% of EC cases exhibited isolated loss of PMS2 expression, a rate significantly higher than the 0.04% observed in CRC. Additionally, 24% of tumors with isolated loss of PMS2 expression were found to carry *MLH1* germline mutations [94]. Recently, Carvalho et al. documented a case of a patient with EEC who exhibited an isolated deletion of the PMS2 protein alongside a germline *MLH1* mutation, c.193G > A (p.Gly65Ser) [95], indicating a potential association between certain PMS2 defects and MLH1 abnormalities. Simultaneously, in the report by Dudley et al., among eight patients with EC who underwent germline examination (without distinguishing pathological types), six carried only *PMS2* pathogenic germline mutations: c.1831_1832insA, c.765C > A, c.1831 insA, c.247_250dupTTAA, c.137G > T (p.S46I), and c.2123delA [94]. Furthermore, Cui et al. identified a patient with EEC who had a heterozygous germline *PMS2* mutation c.1577delA (p.Asp526Alafs* 69), and genetic testing revealed that the patient’s mother and sister carried the same mutation. The patient was diagnosed at age 26, suggesting that *PMS2* mutations may accelerate cancer development [96]. Notably, no data are currently available on somatic *PMS2* mutations in EC, implying that *PMS2* may not be implicated in Lynch-like EC in the same manner as other MMR genes.

### 3.7. EXO1

*EXO1* is located on chromosome 1q43 and was first identified in Schizosaccharomyces pombe [97]. As a pivotal gene within the MMR system, *EXO1* encodes a protein primarily responsible for the excision phase of MMR, facilitating the formation of a mismatch gap [39]. In contrast to the major MMR genes, *EXO1* is classified as an auxiliary gene within the pathway. Emerging evidence indicates that *EXO1* anomalies can impair DNA repair, thereby increasing genomic instability and elevating the risk of EC [98]. Dámaso et al. identified a rare germline mutation, c.2212-1G > A, in *EXO1* in 115 patients with LLS (MMR-deficient tumors but no traditional germline MMR mutations) using NGS and Sanger sequencing. This mutation results in the deletion of six amino acids in the *MSH2* interaction domain (p.Val738_Lys743del) [61]. Similarly, Xavier et al. identified three distinct germline mutations in the *EXO1* gene—c.1928T > A, c.2485G > T, and c.2009A > G—in 274 patients with LLS. These mutations affect the interaction region between *EXO1* and *MLH1*/*MSH2*, with the first two predicted to be pathogenic [88]. Talseth-Palmer et al. identified a germline *EXO1* mutation in 14 patients who met the diagnostic criteria for LS but in whom traditional methods did not detect mutations; IHC showed *MLH1* loss [99]. Singh et al. also detected two patients with EC with *EXO1* germline mutations (pathological subtypes not specified) using NGS. The mutation sites were c.409G > T (p.Ala137Ser) and c.-84T > G, but they were not confirmed to be pathogenic [66]. These findings indicate that *EXO1* germline mutations may contribute to the LS phenotype by impairing MMR function, thereby increasing the risk of EC in patients with LS. In addition, Levan et al. reported that *EXO1* was overexpressed in non-survivors compared with survivors of EEC [100]. Studies have shown that *EXO1* participates in cell cycle regulation via the RB/E2F pathway. Overexpression of *EXO1* may disrupt cell cycle regulation, leading to uncontrolled cell proliferation and promoting tumor progression [100,101]. No studies have reported somatic *EXO1* mutations in patients with EC. In conclusion, germline mutations in *EXO1* may compromise the MMR function by disrupting its interaction with *MLH1*/*MSH2*, thereby contributing to the LS phenotype and increasing the risk of EC. Concurrently, *EXO1* overexpression may induce cell cycle dysregulation through the RB/E2F pathway, promoting tumor progression. These findings suggest that *EXO1* contributes both to genetic susceptibility and to tumor promotion in the development of EC.

## 4. Detection and Diagnosis Process of MSI-H/dMMR Type EC

Based on existing literature, the diagnostic process and technical application of dMMR/MSI EC can be summarized as follows: Before molecular testing, patients at high risk of LS can be identified by detailed family history assessment and clinical features such as early-onset disease (<50 years), metachronous or multiple primary cancers, and LS-compliant tumor lineage [87,102]. The expression of four MMR proteins (MLH1, MSH2, MSH6, and PMS2) is assessed by IHC to screen for the presence of dMMR; complete loss of any protein indicates dMMR [87,103]. Following IHC, MSI status testing is recommended to supplement the results. Studies have shown that IHC and PCR-MSI results are consistent in most cases; however, a proportion of cases remain discordant, highlighting the value of combined testing [104,105]. The combination of IHC and MSI testing has been shown to increase the recognition rate of LS-related tumors to nearly 100% [87]. Traditional MSI detection mainly adopts the PCR-capillary electrophoresis (PCR-CE) method, which is regarded as the ‘gold standard’ for functional testing [105]. A previous study summarized all currently available MSI testing kits based on standard PCR [106]. However, several studies strongly recommend using next-generation sequencing (NGS) for MSI testing [87,102,103]. NGS can accurately evaluate MSI status through dedicated algorithm, detect a large number of microsatellite loci simultaneously, and apply lower threshold to determine MSI-H status in a quantifiable manner, thereby greatly improving sensitivity and specificity [102,103]. A new NGS-based MSI testing method was proposed in a study that reported superior performance compared with previously published methods [15]. In addition, NGS provides a comprehensive approach to simultaneously obtain MSI status, TMB, and specific gene mutation information simultaneously, and is gradually becoming a new standard in clinical laboratories [102,103]. It is necessary to distinguish LS-related from sporadic cases after the discovery of dMMR/MSI-H tumors. If IHC shows MLH1 protein loss, *MLH1* promoter methylation testing should be performed to identify sporadic EC, which is usually caused by *MLH1* promoter methylation [87,102]. Genetic counseling and germline testing should be performed for *MLH1* methylation-negative cases and for dMMR tumors with loss of other MMR proteins (MSH2, MSH6, or PMS2) [87,102]. NGS also plays an important role at this stage. It can reliably detect germline mutations, including nucleotide substitutions, insertions/deletions, and copy number variation (CNV), through multi-gene panel testing, thereby providing comprehensive evidence for the diagnosis of LS [87,102]. At this stage, the traditional diagnostic process can distinguish dMMR/MSI EC into LS-related and sporadic subtypes. Studies have shown that patients with dMMR account for approximately 25–30% of all EC cases, with LS-related EC accounting for approximately 5% [19,21], and sporadic EC accounts for approximately 70% [22].

It is worth noting that a small number of studies have proposed the term ‘Lynch-like’ to describe patients who exhibit MSI/dMMR tumor characteristics (without hypermethylation of the *MLH1* promoter) but have not found pathogenic germline MMR gene mutations [87]. Patient populations with Lynch-like disease are highly heterogeneous and may include undiagnosed germline mutations, biallelic somatic mutations, or other genetic mechanisms [87,107,108,109]. Studies have shown that Lynch-like ECs account for a considerable proportion of dMMR EC, comparable to or even exceeding the prevalence of LS [87,107]. In addition, studies have suggested that differences may exist in the immune microenvironments of LS-related, Lynch-like, and sporadic EC, which may affect their therapeutic effects [110,111]. Current clinical practice relies heavily on traditional screening methods, which may result in the neglect of Lynch-like EC. In addition, once dMMR/MSI status is determined and sporadic EC is identified, NGS-mediated germline testing is not performed, and Lynch-like EC may even be misclassified as LS-related EC, causing unnecessary family screening anxiety as well as wasting medical resources and imposing additional financial burden.

Studies have shown that many patients have undergone somatic mutation testing using NGS technology, and the mutation data obtained provide an important basis for developing subsequent treatment strategies [103]. Therefore, our research supports the further distinction between Lynch-like EC and LS beyond traditional classification, which has significant implications for clinical management, cancer screening strategies, and genetic counseling for patients and their families [87,107]. With the advancement in detection technology, the etiology of Lynch-like EC will become clearer, facilitating personalized management [87,109]: The initial diagnostic approach for Lynch-like EC is the same as that for LS: MMR protein (MLH1, PMS2, MSH2, MSH6). IHC testing should be performed on all EC tissues. The complete absence of any of these proteins is considered indicative of dMMR [87,103]. If MLH1 protein deficiency is detected, *MLH1* promoter methylation testing should be performed. Positive methylation supports the diagnosis of sporadic EC and can largely rule out LS- and Lynch-like cases. Negative methylation results require further evaluation. For patients with MMcR deficiency and negative *MLH1* methylation or MSI-H status and negative *MLH1* methylation, germline sequencing of MMR genes should be conducted [87,102]. When no pathogenic mutations are identified during germline testing, paired tumor and normal tissue NGS should be performed to identify somatic mutations affecting both alleles of the same MMR gene [109]. Subsequently, all patients with suspected LS-related EC are recommended to receive professional genetic counseling to understand genetic risks, family implications, and management strategies [87,102,112]. Genetic counselors play a crucial role in the testing process by guiding patients and family through appropriate testing, interpreting variants of uncertain significance or negative results, and ensuring accurate information transfer and cascade testing to help alleviate testing-related anxiety [112]. The genes commonly tested include *MLH1*, *MSH2*, *MSH6,* and *PMS2*. Additionally, if the association between non-traditional MMR genes (*MSH3*, *MLH3*, *EXO1*) and dMMR/MSI EC is validated through extensive clinical practice or future trials, these genes may be considered for inclusion in gene testing protocol based on specific clinical circumstances (e.g., the presence of MSI status without dMMR, and all traditional MMR genes being normal). In summary, this integrated diagnostic strategy, which combines IHC, MSI testing, molecular typing, and genetic counseling, is expected to enable individualized and precise diagnosis and treatment of EC, ultimately improving patient prognosis and the health management of their families.

## 5. Treatment of MSI-H/dMMR EC

### 5.1. Related Treatment

Presently, the NCCN guidelines integrate TCGA molecular typing into clinical practice, whereas the ESGO guidelines recommend ProMisE to guide the treatment of early EC [113]. ProMisE, a more pragmatic classification system based on TCGA, was proposed by Talhouk et al. (2017) [114]. It includes categories such as *POLE*-mutant (*POLE*-positive), high-instability MSI (H-MSI), MMR-deficient (MMR-d), p53-abnormal (p53abn), and nonspecific molecular profile (NSMP). This system employs MMR IHC instead of MSI testing, enabling rapid genomic classification to aid prognostic determination and treatment guidance [114]. According to NCCN Guidelines version 2.2025, total hysterectomy and bilateral adnexectomy are the primary treatments for EC. For patients with strong fertility needs or high surgical risk, conservative treatment may be used, including oral or intrauterine progesterone (levonorgestrel-releasing intrauterine device) combined with hysteroscopic focal endometrial resection [115]. The NCCN guidelines delineate a set of inclusion criteria for conservative treatment. Several studies have reported that, compared with MMR-skilled (MMRp) EC, dMMR EC exhibit a unique tumor immune microenvironment characterized by abundant expression of neoantigens. Increased infiltration of immune cells in the tumor stroma—such as granzyme B + cells, activated cytotoxic T cells (CTL), PD-L1+ cells—as well as T cell-mediated antitumor immune responses are generally upregulated [116,117]. Under normal physiological conditions, immune tolerance is maintained through the PD-1/PD-L1 checkpoint pathway, which prevents excessive immune activation. The mechanism involves PD-L1 binding to PD-1 on the surface of T cells, transmitting inhibitory signals that reduce T-cell activation and function [118]. Mittica et al. reported that tumor cells can also express PD-L1. Therefore, in the EC tumor microenvironment, tumor cells can bind to PD-1 on infiltrating T cells via surface PD-L1, inhibiting T-cell activity and cytotoxic function, thereby promoting immune evasion [17]. dMMR/MSI-H EC exhibit high PD-1 and PD-L1 expression and show immune escape characteristics [16,119]. Therefore, they usually show high sensitivity to ICI treatment and are excellent candidates for this therapy. Currently, pembrolizumab and dostarlimab are the main immunotherapeutic drugs used in clinical practice. These drugs block the interaction between PD-1 and PD-L1, reactivating T cells and restoring their immune function, thereby enhancing the ability of the immune system to recognize and kill tumor cells and exert anti-tumor effects [117]. Both drugs have been approved by the US Food and Drug Administration (FDA) for the treatment of patients with advanced or recurrent MSI-H/dMMR EC [120,121]. Recent studies have shown that immunotherapy combined with chemotherapy can significantly improve the overall survival (OS) and progression-free survival (PFS) in patients with dMMR compared with chemotherapy alone [122]. Moreover, the NCCN clinical guidelines recommend pembrolizumab/dostarlimab combined with paclitaxel and carboplatin as the first-line standard treatment strategy for patients with advanced/recurrent MSI-H/dMMR EC [123]. Furthermore, some patients exhibiting *MLH1* hypermethylation demonstrate increased expression of estrogen and progesterone receptors (ER/PR), for whom hormone therapy is recommended [111]. Another study reported that *MLH1* may activate the MLH1/c-Abl apoptosis signaling pathway, thereby enhancing the sensitivity of endometrial cancer (EC) cells to cisplatin and promoting apoptosis. Conversely, the knockout of *MLH1* decreases the sensitivity of EC cells to cisplatin. Thus, modulating the expression of *MLH1* could potentially regulate the sensitivity of EC cells to cisplatin, thereby influencing therapeutic outcomes [124].

### 5.2. Noteworthy Issues in Treatment

#### 5.2.1. Immune Microenvironment Characteristics and Therapeutic Effect Differences Among Different dMMR Subtypes of EC

At the same time, the NCCN guidelines currently regard dMMR as a homogeneous group, and no independent scheme has been developed for dMMR from different sources. Recent studies have shown the value of distinguishing between three sources of EC: although both have high levels of MSI, hereditary and sporadic EC have different immune microenvironments. For example, the CD8+ T cell count of LS-related EC is significantly higher than that of sporadic EC, while the number of CD68+ macrophages and PD-L1+ macrophages in the latter is significantly higher than that in the former, but LS-related tumors are more likely to express PD-L1 [110,116,125,126]. Compared to patients with sporadic EC, the infiltration of CD3+ T cells in patients with Lynch-like EC showed an increasing trend; however, the infiltration of CD68+ macrophages was significantly higher [127]. It is worth noting that high levels of tumor infiltration by CD3+ and CD8+ T cells have been confirmed in multiple studies to predict significantly better therapeutic responses and survival benefits against PD-1/PD-L1 immunotherapy [128,129]. Compared with Lynch-like EC, the immune microenvironment of sporadic EC is characterized by low PD-L1 positive rate, lower T cell inflammation score, and interferon γ score [111]. In addition, the CD8+ T cell count in the tumor compartment of LS-associated EC is significantly higher than that in the Lynch-like EC group [125]. In summary, the number of CD8+ T cells in the LS-related germline mutation (EC) matrix is large, the number of CD68+ and PD-L1+ macrophages in the matrix and tumor compartment is small, and the immunogenicity is strong [110,116,125,126]; the number of CD8+ T cells in the stroma of sporadic (MLH1 methylated) EC is relatively small, the number of CD68+ and PD-L1+ macrophages in the stroma and tumor compartment is relatively large, and the infiltration of CD3+ T cells is relatively low [110,116,125,126,127]. Lynch (somatic mutation) EC have relatively few CD8+ T-cells, CD3+ T-cell infiltration shows an increasing trend, and CD68+ macrophage infiltration is significantly higher [125,127] (Figure 2). This indicates that different EC sources respond differently to the same ICI. Similarly, after receiving Pembrolizumab treatment, in the study of Bellone S et al., the objective remission rates of Lynch-like EC and sporadic recurrent EC patients were 100% and 44%, respectively, while the 3-year PFS and OS ratios were 100% and 30% and 100% and 43%, respectively [127]. Bogani et al. reported that the OS of patients with *MLH1* hypermethylated EC was significantly shorter than that of patients with *MLH1* mutations [122]. In a study by Ozawa et al., the overall remission rate of sporadic EC was 75%, Lynch-like EC and LS-related EC were both 100% (one patient with complete remission each), and PFS and OS were longer than sporadic EC, is suggested that pembrolizumab monotherapy may be more effective in Lynch-like and LS-related groups [130]. However, limited studies are comparing the efficacy of the three subtypes, especially between Lynch-like EC and LS-related EC. According to the above differences in the tumor invasion microenvironment, in the context of large-sample studies, it is predicted that the efficacy of pembrolizumab monotherapy for Lynch-like EC should be between sporadic EC and LS-related EC.

#### 5.2.2. Discussion on the Mechanism of Immunotherapy Resistance

It is worth noting that studies have shown that more than 50% of MSI-H patients do not respond to ICIs, and this drug-resistance phenomenon may result from multiple mechanisms working together. Research has shown that in *MLH1*-methylated EC, the frequency of *JAK1* loss-of-function mutations is significantly increased, and this mutation has been confirmed as one of the key molecular mechanisms leading to primary resistance to ICIs [111,131]. The mechanism mainly stems from the disruption of the IFN-γ signaling pathway by *JAK1* mutations, which manifests as blocked STAT1 phosphorylation, downregulated expression of antigen-presenting molecules (such as MHC-I), and reduced production of pro-inflammatory factors, ultimately leading to tumor immune escape [131,132]. Stress factors in the tumor microenvironment, such as endoplasmic reticulum stress, can also activate the *JAK1*-STAT3 pathway through a PERK-dependent pathway, further exacerbating the formation of a local inflammatory and immunosuppressive microenvironment [133]. Additionally, β2-microglobulin (B2M) is a key component in the correct assembly and antigen presentation process of MHC-I molecules and plays a core role in mediating the presentation of tumor antigens to CTLs and initiating specific immune responses [133]. Walkowska J et al. conducted IHC analysis on 169 cases of LS CRC and found that the deletion rate of *B2M* was 28%; they proposed that *B2M* deletion may reduce the sensitivity of LS-related CRC to PD-1 inhibitor treatment [134]. Liu F et al. further studied the *B2M* mutation characteristics in three MSI-H/dMMR high-incidence cancers, including EC, and the results showed that the *B2M* gene mutation rate in EC reached 13.6%. This study also indicated that in MSI-H/dMMR tumors, patients with B2M mutations had a higher TMB (*p* = 0.026) than wild-type patients, indicating that B2M mutations may further promote the accumulation of mutations [135]. The above results suggest that some MSI EC may be resistant to ICIs due to abnormal B2M function, which leads to the obstruction of the antigen presentation process mediated by MHC-I molecules, preventing CD8+ T cells from effectively recognizing tumor neoantigens and inhibiting the initiation of effective anti-tumor immune responses.

Previous studies have shown that hypermethylation of the *MLH1* promoter can lead to a highly immunosuppressive microenvironment in various tumors, including EC [116,126]. In *MLH1*-hypermethylated EC, the infiltration levels of CD68+ and PD-L1+ macrophages were higher than those in LS-related EC. At the same time, a large number of regulatory T cells (Tregs) and myeloid-derived suppressor cells (MDSCs) are also common in these tumors, which together lead to decreased sensitivity to PD-1/PD-L1 monotherapy [126,136,137,138,139]. In addition, multiple studies have confirmed that immunosuppressive cells such as MDSCs, tumor-associated macrophages (TAMs), and Tregs are generally enriched in the microenvironment of MSI-type tumors [18,140]. These cells collectively form a highly immunosuppressive microenvironment, creating favorable conditions for tumor cells to evade immune surveillance and promote tumor growth and progression [141]. Under physiological conditions, immature myeloid cells proliferate normally, differentiate into various types of mature myeloid cells, and do not possess immunosuppressive functions. However, under pathological conditions, a variety of tumor-derived factors interfere with the normal differentiation process, leading to the stagnation of such cells in the immature stage, which in turn are recruited to the tumor microenvironment and differentiated into MDSCs [142]. Research indicates that MDSCs can induce immune tolerance by suppressing T-cell function and proliferation, while also directly supporting tumor growth and progression through mechanisms such as promoting angiogenesis and matrix deposition [143]. Tregs impede T cell activation by inhibiting the antigen-presenting function of dendritic cells (DCs), and directly suppress the proliferation and function of CD4+ and CD8+ T cells by secreting inhibitory cytokines, thereby downregulating anti-tumor immune responses [144]. Macrophages can be divided into the M1 and M2 subtypes according to their functional characteristics. M1 macrophages typically participate in inflammatory responses and pathogen clearance, thereby promoting the killing of tumor cells. In contrast, M2 macrophages exert anti-inflammatory effects and promote tissue repair; within the tumor microenvironment, they are often recruited to support tumor growth [145]. TAMs tend to have an M2-like phenotype in most cases, and their high infiltration levels (evaluated by CD68+ staining) are closely related to poor patient prognosis [146,147]. Recent studies have found that somatic mutations in nontraditional MMR genes can also influence the immune microenvironment of MSI tumors [148,149]. In a variety of cancers, the high expression of *EXO1* is positively correlated with the expression of most immunomodulatory genes (including chemokines, receptors, MHC molecules, and immune stimulation/inhibition molecules) and immune checkpoint molecules (such as CD276 and HMGB1) [148]; however, the high expression of *EXO1* was significantly negatively correlated with the overall level of immune infiltration, suggesting that there may be an immunosuppressive microenvironment. Further analysis revealed that *EXO1* expression was positively correlated with the infiltration of immunosuppressive or specific helper cell subsets, such as MDSCs, follicular helper T cells, and neutrophils, and negatively correlated with the infiltration of antitumor immune cells, such as NK cells. These findings indicate that *EXO1* plays a dual role in regulating immune responses within the tumor microenvironment, potentially influencing both immune evasion mechanisms and the recruitment or regulation of specific immune cell populations. Furthermore, Xu et al. revealed through bioinformatics analysis that *MSH3* expression levels were significantly associated with immune cell infiltration in the renal cell carcinoma (RCC) tumor microenvironment. Low expression of *MSH3* is negatively correlated with Treg enrichment, suggesting that it may influence the formation of an immunosuppressive microenvironment [149]. The expression of *MSH3* correlates with the expression levels of multiple immune checkpoint genes, which further indicates that it may play a role in regulating the tumor immune response. These data suggest that the non-classical MMR genes *EXO1* and *MSH3* may participate in tumor progression and immune evasion mechanisms by influencing the composition and function of immune cells within the MSI tumor microenvironment.

Presently, chemotherapy drugs such as cisplatin are used as adjuvant treatment, and their application may affect the efficacy of ICIs. Cisplatin, a classic first-generation chemotherapeutic drug, is widely used to treat various malignant tumors [150]. It binds to the DNA of tumor cells, disrupts their structure and function, and causes severe DNA damage, thereby inhibiting the growth and proliferation of tumor cells [151]. Li et al. reported that DNA interstrand cross-linking (ICLs) is a serious form of DNA damage that can be induced by exogenous agents such as cisplatin, and its repair often depends on the homologous recombination (HR) pathway [152]. Studies have shown that, in addition to participating in the MMR pathway, *MLH3* and *PMS2* provide essential nuclease activity for the HR pathway, promote the processing of recombinant intermediates, ensure efficient repair of cisplatin-induced DNA damage, and reduce damage accumulation as well as apoptosis or proliferation arrest. Studies have shown that when mutations occur in *MLH3* or *PMS2*, the function of the HR pathway is impaired, and cisplatin-induced DNA damage cannot be effectively repaired. The continuous accumulation of damage will further exacerbate genomic instability and trigger apoptosis [18,153]. This indicates that *MLH3* or *PMS2* mutations not only cause dMMR/MSI-H but also affect other critical cellular signaling pathways or DNA damage response mechanisms. These pathway abnormalities, aside from MMR, may be independent of the immune activation mechanism related to dMMR, thereby resulting in tumors with MSI status that are insensitive to anti-PD-L1 treatment. Furthermore, some studies have indicated that these chemotherapy drugs, in addition to directly killing cancer cells, may promote the death of immunogenic cells and indirectly activate anti-tumor immunity [18,154]. Therefore, these drugs are often associated with severe toxic side effects and tumor resistance in clinical settings [155]. Combination regimens of chemotherapy drugs such as cisplatin and ICIs have been widely adopted for the treatment of MSI-H/dMMR ECs. Although dMMR EC usually have a highly immunogenic tumor microenvironment, the toxic effects of chemotherapy drugs may lead to immune cell cycle arrest and even cell apoptosis [156].

In addition, the methylation status of MLH1 is often associated with high ER expression. Studies have shown that the activation status of *JAK1* (such as *p*-JAK1) is significantly correlated with the expression of ER [157], and that the JAK/STAT signaling pathway is widely involved in estrogen-mediated biological processes, including the regulation of cell proliferation and senescence [158,159]. Therefore, in the context of *MLH1* methylation, *JAK1* mutations may affect the sensitivity of tumors to hormone therapy and the response process by altering the cross-dialog between the ER and JAK/STAT pathways. In patients with *MLH1* methylated EC, *JAK1* status can serve as an important biomarker for predicting immunotherapy resistance and evaluating the potential benefits of hormone therapy.

In summary, resistance to ICIs in dMMR/MSI-H EC may represent a complex biological process involving multiple factors and pathways, including genomic mutations, epigenetic regulation, the tumor microenvironment, and therapeutic interventions. Currently, TMB and PD-L1 expression are important predictive indicators. A high TMB level indicates the generation of more neoantigens and enhanced immunogenicity. High PD-L1 expression indicates preexisting immune responses, and both help screen potential beneficiary groups of ICIs. Studies have shown that *JAK1* and *B2M* mutations/deletions mediate primary drug resistance by disrupting the IFN-γ signaling pathway and antigen presentation mechanism, respectively. In addition, the immune composition of the tumor microenvironment (such as the enrichment of Tregs, MDSCs, and TAMs) and the immunosuppressive ecosystem shaped by the hypermethylation phenotype of the *MLH1* promoter are key factors that lead to a poor response. ER expression may also affect the treatment response through its interaction with the JAK/STAT pathway. Based on this, future clinical management may focus on integrating multidimensional biomarkers (such as MSI-H, TMB, PD-L1, JAK1, B2M, tumor microenvironment classification, and ER status) to identify potential high-risk populations for drug resistance and guide the formulation of treatment strategies. Existing data show that pembrolizumab monotherapy has a significant effect on patients with LS-related EC and Lynch-like EC and can induce durable remission and even potential cure. Therefore, patients with this subtype should be prioritized. For most patients with sporadic EC, as they often have an immunosuppressive tumor microenvironment (TME) and a limited response to monotherapy, it is necessary to actively explore combination treatment strategies, such as ICIs combined with JAK inhibitors, chemotherapy, hormone therapy, or drugs targeting the TME, to reverse the drug-resistant microenvironment. When designing combination regimens, emphasis should be placed on optimizing drug combinations, administration sequencing, and treatment cycles, while rigorously monitoring and managing additive toxicity. Currently, in addition to pembrolizumab, dostarlimab monotherapy is also a treatment option for dMMR/MSI-H EC. Lenvatinib combined with pembrolizumab can be considered for patients who do not respond to pembrolizumab monotherapy. In conclusion, through subpopulation stratification guided by markers and the optimization of treatment strategies, more precise individualized treatments can be achieved, ultimately improving the prognosis of patients.

#### 5.2.3. dMMR Status and Poor Response to Progesterone Treatment

Patients with dMMR exhibit lower ER/PR expression, and their response to progesterone therapy may be poor [160]. Research focusing on young patients with early-stage EC indicates that the prevalence of dMMR in this demographic ranges from approximately 7% to 34% [160,161,162,163]. Two retrospective studies comparing the efficacy of progesterone treatment in early premenopausal patients with dMMR versus those without dMMR revealed remission rates of 0% to 44.4% in the dMMR cohort, compared with 53% to 82.2% in the non-dMMR cohort. This disparity underscores the significantly diminished response to progesterone therapy in patients with dMMR [163,164]. Therefore, some researchers consider dMMR a potential marker of early progesterone treatment failure [163,165]. Currently, dMMR has not been definitively established as a biomarker of conservative treatment in patients with early-stage disease. It is crucial to acknowledge that patients who do not respond to progesterone and wish to preserve fertility require novel, conservative treatment options. Although ICIs have demonstrated favorable efficacy in patients with advanced or recurrent dMMR EC, their safety and efficacy in early-stage EC remain undetermined. The clinical trial NCT06278857, projected to conclude in 2027, aims to investigate the efficacy, safety, and impact of dostarlimab on fertility in women with early-stage dMMR EEC. This trial is anticipated to provide a novel therapeutic option for these patients and to facilitate fertility preservation in women of reproductive age [166]. However, the trial enrolled only 10 patients who met the inclusion criteria, highlighting the necessity for larger prospective studies to further explore fertility outcomes in young patients with dMMR and early-stage EC. Additionally, comparative analyses of the efficacy and safety of various inhibitors in these patients are warranted.

#### 5.2.4. Possible Trends in Optimizing Diagnostic Strategies

The above research suggests that promoting NGS-based MSI testing and further differentiating the three dMMR subtypes may help formulate more precise treatment plans for patients with EC. NGS can simultaneously provide information on MSI status, germline mutations, and somatic mutations, which is particularly useful for identifying Lynch-like ECS. However, the current cost of NGS is relatively high, and its widespread implementation in clinical practice remain challenging. We suggest that, in medical institutions where economic and technical conditions permit, NGS should be prioritized to achieve more comprehensive molecular typing and optimize treatment strategies. However, all the above findings require further prospective studies or retrospective analyses to verify, including investigations of differences between LS-related and sporadic EC immunotherapy, differences between LS-related and Lynch-like EC immunotherapy, and novel MSI detection methods based on NGS.

## 6. Summary and Outlook

**Figure 3:**
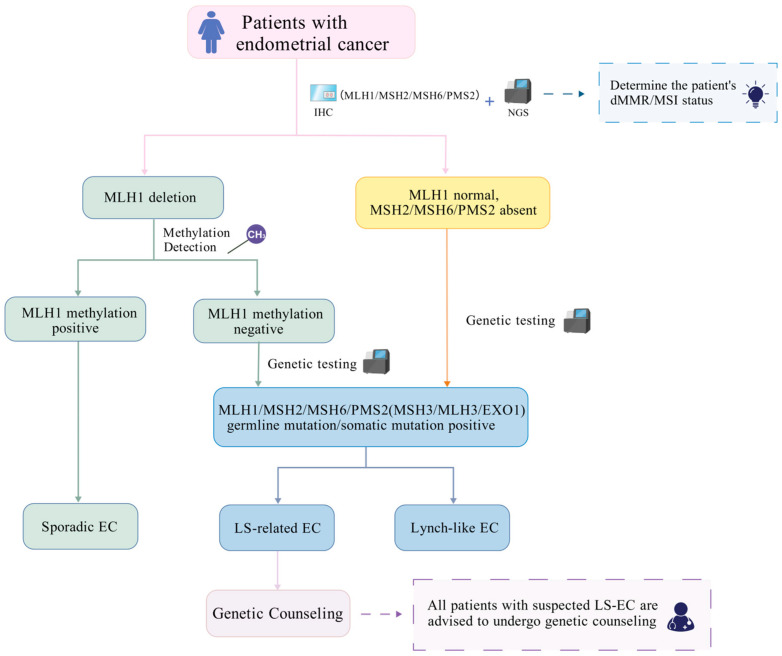
Optimized MSI-H/dMMR EC screening process. First, the MMR proteins MLH1, MSH2, MSH6, and PMS2 were detected by IHC, and the complete deletion of any protein was determined as dMMR. (1) If MLH1 is absent, MLH1 promoter methylation detection should be conducted. If the result is positive, sporadic EC should be diagnosed. If the result is negative, germline testing should be conducted. If no pathogenic mutation is found in the germline testing, NGS testing pairing tumor tissue with normal tissue should be performed to distinguish LS-related EC from Lynch-like EC. (2) If MLH1 is normal, but another MMR protein (MSH2, MSH6, PMS2) is absent or MSI-H status is detected, system testing should be performed. If system testing finds no pathogenic mutations, tumor tissue and normal tissue matching NGS should be conducted. In addition, for patients suspected of having LS EC, it is recommended that they receive professional genetic counseling to understand the genetic risk, family influence, and subsequent management strategies. Created with BioGDP.com [43].

The molecular characterization of EC has significantly transformed its diagnostic and therapeutic approaches. Notably, the MSI-H/dMMR subtype has emerged as a critical target for immunotherapy, owing to its distinct immune microenvironment. This review systematically examines the roles of core MMR pathway genes (*MLH1*, *MSH2*, *MSH6*, *PMS2*) and auxiliary genes (*MSH3*, *MLH3*, *EXO1*) in EC. We elucidated the molecular mechanisms leading to MSI and genomic instability through dMMR and highlighted the necessity for novel therapeutic strategies for young patients of childbearing age with dMMR who strongly desire to conceive. The heterogeneity of dMMR subtypes from different sources (hereditary, sporadic, and Lynch-like) in terms of immune microenvironment, treatment response, and prognosis has been explored in detail, and the potential mechanisms of drug resistance in some patients have been extensively investigated. Current studies indicate that both germline and somatic mutations in *MSH2*, *MSH6*, and *MLH1*, including those associated with aggressive histological variants, are linked to EC, whereas abnormalities in *MSH3*, *MLH3,* and *EXO1* may indirectly facilitate tumor progression through genomic instability or cell cycle dysregulation (Table 1). Although ICIs have markedly enhanced survival outcomes in patients with advanced dMMR, approximately 50% of these patients remain unresponsive. Current guidelines that treat dMMR subtypes uniformly may neglect the potential benefits of personalized therapy. Consequently, future research must achieve breakthroughs in several key areas. First, it should promote the routine implementation of NGS technology to accurately distinguish germline mutations, somatic mutations, and epigenetic abnormalities, thereby optimizing the diagnostic process and improving the detection rate of EC (see Figure 3). Second, it is essential to validate the role of auxiliary MMR gene abnormalities, such as *MSH3*, *MLH3*, and *EXO1*, in EC to support the expansion of MMR gene screening. Third, prospective studies with large sample sizes should be conducted to compare the sensitivity of dMMR subtypes of various origins to ICIs, chemotherapy, and targeted therapies, with the aim of establishing individualized treatment pathways based on molecular profiling. Lastly, emphasis should be placed on fertility preservation for young patients with dMMR, necessitating the development of treatment protocols that balance therapeutic efficacy with reproductive function preservation. Through interdisciplinary collaboration and technological innovation, the diagnostic and therapeutic system for EC is anticipated to advance significantly, ultimately enhancing patient survival rates and quality of life.

## Figures and Tables

**Table 1:** The function of MMR genes in MSI-H/dMMR EC.

Gene	Location	Protein Function	EC Correlation Anomaly	EC Linked Data	Clinical Features
MSH2	2p21-p16.3	It interacts with MSH6 to form the MutSα complex, which is responsible for recognizing smaller mismatched bases and initiating the MMR process [35].	Germline mutation Somatic mutation	Germline mutations contribute to approximately 32% of LS [46].	Germline mutation: CCEC, USC, uterine carcinosarcoma [48] Somatic mutation: atypical hyperplasia/endometrial intraepithelial neoplasia, EEC, USC [49,50]
MSH6	2p15-16	It forms a complex with MSH2, known as MutSα, to recognize subtle base pair mismatches and initiate the MMR process [35].	Germline mutation Somatic mutation	Germline mutations contribute to approximately 19.4% of LS [53].	Germline mutation: USC [57] Somatic mutation: EEC [58,60]
MSH3	5q14.1	It forms a complex with MSH2, known as MutSβ, to recognize large, mismatched bases and has overlapping functions with MSH6 [35,63,64].	Germline mutation Somatic mutation	-	Germline/somatic mutation: EEC, USC [25] Somatic mutation: endometrial hyperplasia [67]
MLH1	3p22.2	The MutLα and MutLγ complexes are formed through their association with PMS2 and MLH3, respectively [70].	Germline mutation Somatic mutation Methylation	Germline mutations contribute to approximately 42% of LS [77]. Methylation contributes to approximately 70% of dMMR EC [71,72,73].	Germline mutation: EEC [48]
MLH3	14q24.3	It interacts with MLH1 to form the MLH1-MLH3 (MutLγ) complex, which primarily functions during meiosis. Additionally, it serves a complementary role alongside PMS2 in the process of MMR [85,86].	Germline mutation Somatic mutation	-	Germline/somatic mutation: EEC, CCEC, USC [24,25,26]
PMS2	7p22.1	It interacts with MLH1 to form a complex known as MutLα, which possesses endonuclease activity [36].	Germline mutation	Germline mutations contribute to approximately 6% of LS [92].	Germline mutation: EEC [94,95,96]
EXO1	1q43	Participate in the MMR resection step to promote the formation of mismatched directional gap [39].	Germline mutation	-	-

## Data Availability

No new data were created.

## Abbreviations

The following abbreviations are used in this manuscript:

CCEC	clear cell endometrial carcinoma
CE	capillary electrophoresis
CNV	copy number variation
CRC	colorectal cancer
dMMR	deficient mismatch repair
EC	endometrial cancer
EEC	endometrioid endometrial carcinoma
ESGO	European Society of Gynecological Oncology
FIGO	International Federation of Gynecology and Obstetrics
HNPCC	hereditary non-polyposis colorectal cancer
HR	homologous recombination
H-MSI	high-instability microsatellite instability
IHC	immunohistochemistry
ICIs	immune checkpoint inhibitors
ICLs	interstrand cross-linking
LLS	Lynch-like syndrome
LS	Lynch syndrome
MDSCs	myeloid-derived suppressor cells
MLH1	mutL homolog 1
MLH3	mutL homolog 3
MMR	mismatch repair
MMR-d	mismatch repair deficient
MSH2	mutS homolog 2
MSH3	mutS homolog 3
MSH6	mutS homolog 6
MSI	microsatellite instability
MSI-H	high microsatellite instability
MutLα	MLH1-PMS2
MutLγ	MLH1-MLH3
MutSα	MSH2-MSH6
MutSβ	MSH2-MSH3
NCCN	National Comprehensive Cancer Network
NGS	next-generation sequencing
NSMP	non-specific molecular profile
OS	overall survival
PCR	polymerase chain reaction
PCR-CE	polymerase chain reaction-capillary electrophoresis
PCNA	proliferating cell nuclear antigen
PD-1	programmed death receptor 1
PD-L1	programmed death ligand 1
PMS2	postmeiotic segregation increased 2
POLE	polymerase epsilon
ProMisE	Proactive Molecular Risk Classifier for Endometrial Cancer
RCC	renal cell carcinoma
RFS	recurrence-free survival
RFC	replication factor C
RPA	replication protein A
ssDNA	single-stranded DNA
TAMs	tumor-associated macrophages
TCGA	The Cancer Genome Atlas
TILs	tumor-infiltrating lymphocytes
TMB	tumor mutational burden
TME	tumor microenvironment
USC	uterine serous carcinoma
WES	whole-exome sequencing
